# Supporting Radiology Resident Education and Clinical Decision-Making With Large Language Models: Comparative Study of Reasoning Models DeepSeek-R1 and ChatGPT-o1

**DOI:** 10.2196/86974

**Published:** 2026-06-26

**Authors:** Semil Eminovic, Robin Schmidt, Bogdan Levita, Maximilian Lindholz, Anna-Maria Haack, Alina Burdenski, Maurice Bui, Isabel Theresa Schobert, Andrea Dell’Orco, Jawed Nawabi, Tobias Penzkofer

**Affiliations:** 1 Department of Radiology, Charité - Universitätsmedizin Berlin, Augustenburger Platz 1, Berlin, 13353, Germany,; 2 Berlin Institute of Health, Charité - Universitätsmedizin Berlin, Berlin, Germany; 3 Department of Neuroradiology, Charité - Universitätsmedizin Berlin, Berlin, Germany

**Keywords:** education, medicine, radiology, artificial intelligence, deep learning, large language models

## Abstract

**Background:**

Radiology trainees require efficient, accurate, and accessible resources to master complex imaging techniques and identify findings that guide clinical decision-making. Large language models (LLMs) are emerging as promising tools for medical education and clinical workflows, offering the potential to enhance learning by providing instant feedback, aiding in diagnostic accuracy, and offering personalized learning experiences. However, systematic comparisons of LLMs for radiology education and clinical support remain limited, particularly regarding differences across subspecialties and resident experience levels.

**Objective:**

This study aimed to evaluate and compare the response quality of 2 state-of-the-art reasoning-based LLMs, namely DeepSeek-R1 and ChatGPT-o1, as clinical and radiology residency support tools, comparing performance across clinical and didactic dimensions, including text- and image-based responses.

**Methods:**

Overall, 27 radiology questions covering 9 radiological subspecialties were answered by both LLMs. Additionally, 6 image-based questions were presented only to ChatGPT-o1 due to its image processing capabilities. Responses were independently rated by 7 radiology residents (postgraduate years 2‐5) across 9 rating criteria grouped into 3 dimensions (factual accuracy, clinical practicality, and didactic value), using a 5-point Likert scale. Statistics compared LLMs, reader experience, and response types for text- as well as image-based for ChatGPT-o1 queries.

**Results:**

DeepSeek-R1 consistently outperformed ChatGPT-o1 across all rating dimensions, with highly significant differences across all criteria (mean ratings: DeepSeek-R1 4.51, SD 0.73 vs ChatGPT-o1 3.73, SD 0.98; *P*<.001). In an exploratory subspecialty-level analysis, DeepSeek-R1 descriptively outperformed ChatGPT-o1 across all subspecialties. For both LLMs accumulated, junior residents tended to rate slightly higher than seniors in 7 of 9 criteria, although differences were not statistically significant. However, for ChatGPT-o1, junior residents rated significantly higher in overall score across all criteria (juniors 3.81, SD 0.64 vs seniors 3.63, SD 0.65; *P*=.02). Image-based responses by ChatGPT-o1 scored significantly lower than text-based (mean 3.19, SD 1.42; *P*=.007), particularly in factual accuracy (mean 2.75, SD 1.45; *P*<.001) and clinical practicality (mean 3.11, SD 1.47; *P*=.03).

**Conclusions:**

Both DeepSeek-R1 and ChatGPT-o1 demonstrate promising potential on simulated radiology question sets designed for educational and clinical contexts, with DeepSeek-R1 outperforming ChatGPT-o1 across all evaluated criteria. These results emphasize the value of open-source models for educational use and provide early evidence that LLMs may support radiology resident training under controlled conditions; however, their real-world educational and clinical effects require further investigation. Future research should prospectively evaluate how LLMs can be integrated into radiology training, assess their impact alongside conventional teaching methods, and investigate multimodal capabilities to better reflect realistic clinical scenarios.

## Introduction

Radiology residents face an exceptionally wide range of organ systems, modalities, and clinical routine and emergency scenarios. This complexity results in a steep learning curve requiring the integration of medical knowledge, image interpretation, and clinical reasoning. Although being in training, these demands collide with the high pace of modern clinical workflows resulting in an increasing workload for radiology residents, which limits their direct supervision or attending consultation within clinical routines [1-3]. Consequently, trainees must frequently retrieve accurate, domain-specific knowledge independently. Conventional educational tools, such as textbooks or general-purpose online platforms, often provide voluminous or poorly targeted results, which may not be considered appropriately in real-life situations where immediate guidance is required. Ultimately, this may lead to frustration, exacerbate cognitive load, and contribute to burnout, which is a common issue faced by radiology residents [4-6].

Recent advances in large language models (LLMs) have prompted growing interest in their potential as educational aides in medicine and radiology [7-11], since they can condense complex topics into concise, context-aware explanations and support iterative follow-up questions that can mimic a human mentor supporting not only point-of-care decision-making but also dedicated study. Additionally, in terms of radiology-specific education, LLMs can integrate image analysis with text-based reasoning to guide learners in correlating imaging data with clinical context. Furthermore, the user may engage in interactive case-based discussions or simulate tumor boards and simply create learning material. However, besides their versatile use cases, LLMs pose a range of risks such as biased, incomplete, or hallucinated responses, which demand robust validation before clinical deployment. While LLMs have yet been proven to perform well on general medical benchmarks, there is an unmet clinical need to explore their potential for educational use in specialty-specific scenarios. To address this gap, this study systematically compared 2 state-of-the-art reasoning LLMs, namely OpenAI’s ChatGPT-o1 [12,13] and DeepSeek-R1 [14], using a structured set of clinically relevant questions that commonly arise during residency. Thereby, both image-description–based diagnostic prompts and knowledge-based questions were included, covering both quick in-shift use cases as well as dedicated study scenarios. The primary comparison in this study focuses on text-based questions that both models could process, whereas the image-based tasks administered only to ChatGPT-o1 were analyzed as an exploratory extension. In summary, this study aims to (1) assess the accuracy, clinical, and didactic value of LLM responses and (2) compare performance between models across clinical and didactic criteria for different radiology subspecialties, thereby providing early evidence on how such models perform on simulated tasks that are relevant for radiology residency training and clinical decision-making. The findings are intended to guide future prospective studies evaluating safe and effective integration of such models into clinical and dedicated educational practice.

## Methods

### General Study Design

Due to the human-generated sample dataset, it was not mandatory to obtain a positive ethics vote from the institute’s ethics committee. In this prospective controlled evaluation study, a set of 33 questions was derived from frequently encountered clinical scenarios in collaboration with both a senior radiologist (TP) with over 15 years of professional clinical and teaching experience and a neuroradiologist (JN) with over 8 years of professional clinical and teaching experience. The generated questions were submitted to the 2 state-of-the-art reasoning LLMs DeepSeek-R1 (DeepSeek, accessed via public web interface on March 28, 2025) and ChatGPT-o1 (full o1 release; OpenAI, accessed via public web interface on March 28, 2025). The LLM-generated responses were independently evaluated by 7 radiology residents (n=4 in their second year of residency: RS, ML, AB, MB; n=1 in third year: AMH; n=2 in fifth year of training: ITS, BL). Because DeepSeek-R1 does not support image inputs, the core model comparison is restricted to text-based questions. The 6 image-based questions presented only to ChatGPT-o1 were therefore treated as an exploratory extension. DeepSeek-R1 and ChatGPT-o1 were selected based on their state-of-the-art performance in both general-purpose reasoning tasks and domain-specific applications [15] and, in particular, because they are well-known among the general public, including radiology residents. Both LLMs use “chain-of-thought” reasoning [16,17] and are explicitly positioned as reasoning-oriented models rather than purely instruction-following systems. They further reflect complementary development paradigms, with DeepSeek-R1 offered as an open-weight model and ChatGPT-o1 as a proprietary system, and both are readily accessible through public interfaces, supporting evaluation in realistic educational settings.

### Ethical Considerations

This study evaluated large language model outputs and did not involve patient data or human participants in research. Based on institutional policy, formal ethics approval was therefore not required. Questions were designed from clinical experience, not individual cases. Raters participated voluntarily, no personal data were collected, and no compensation was provided. All images were sourced from Radiopaedia.org, an open-access educational platform providing fully de-identified cases.

### Question Design and Prompting

A total of 33 questions (27 text-based and 6 image-based) were prepared in the German language covering 9 radiology subspecialties (thoracic, abdominal, oncological, cardiovascular, emergency, head and neck, and musculoskeletal imaging, neuroradiology, and interventional radiology). Each subspecialty included 1 question assessing declarative knowledge and 2 questions describing imaging findings that require diagnostic reasoning. Only ChatGPT-o1 received the 6 image‐based prompts, as it was the sole image‐processing capable model.

Examples of knowledge-based questions (translated):

What are five typical signs of active tuberculosis on CT?

Examples of diagnostic and image-description–based questions (translated):

On magnetic resonance imaging (MRI) there is an extensive bone-marrow edema in the medial tibial plateau without fracture line. There is also no trauma. The patient has load-dependent pain. What is the most likely diagnosis and what are the three most important differential diagnoses?

Example of image-based questions with the corresponding image attached (translated):

Please describe the radiological findings on this MRI image. What is the most likely diagnosis? Which three important differential diagnoses should be considered?

**Figure 1.**
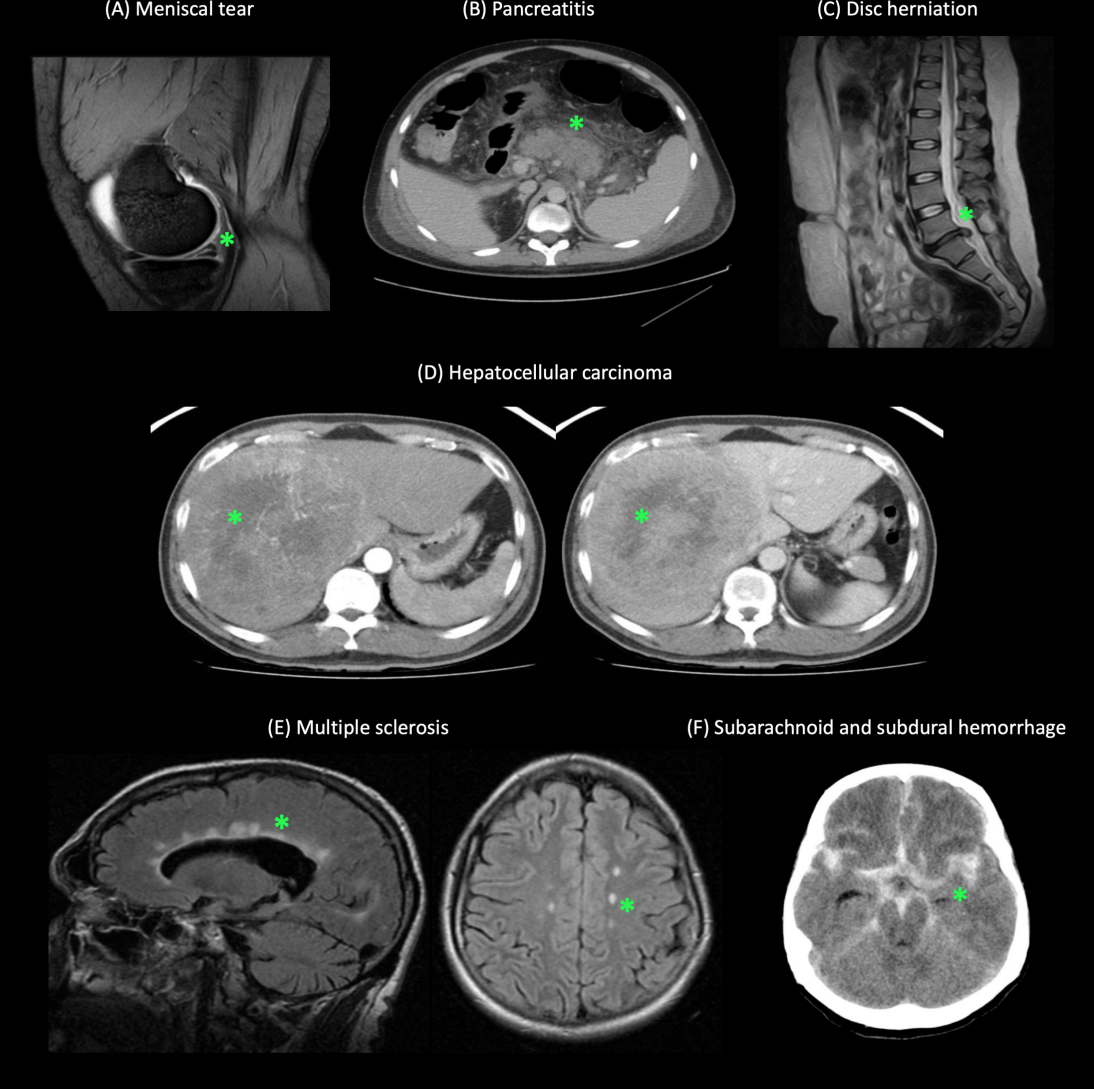
Diagnostic images used as input for the 6 image-based questions submitted to model ChatGPT-o1. Green asterisks are added for illustration only and mark the relevant pathological findings: (A) meniscal tear [18], (B) pancreatitis [19], (C) disc herniation [20], (D) hepatocellular carcinoma [21], (E) multiple sclerosis [22,23], and (F) subarachnoid and subdural hemorrhage [24]. These images are published under Creative Commons Attribution-NonCommercial-ShareAlike 3.0 Unported [25].

Each question was submitted as an independent, standalone prompt in a newly initiated chat session via the public web interface of each LLM (on March 28, 2025), using identical phrasing to ensure comparability and consistency. A new cache-cleared session was started for each query to prevent any context from prior interactions. No few-shot examples were provided, simulating typical resident interactions with LLMs during clinical decision-making and self-directed study. For image-based questions, a corresponding diagnostic image (provided as a static visual reference) was included with each prompt (Figure 1). For illustrative purposes, green asterisks were added to the images in Figure 1 to indicate the key pathological regions, while the unannotated versions were provided to both the raters and ChatGPT-o1.

The complete list of questions is available in the supplementary material (Multimedia Appendix 1). All corresponding model responses are documented as well and available on request.

### Response Evaluation

Responses were rated using a 5-point Likert scale across 9 criteria grouped into 3 dimensions: factual accuracy (correctness, completeness, and precision), clinical practicality (comprehensibility, clinical usefulness, and trustworthiness), and didactic value (explanation depth, structure, and learning facilitation). Each rating dimension represents the average of the scores of its 3 underlying rating criteria (full criteria definitions in Multimedia Appendix 2). To support consistent scoring, reference answers were provided for all 9 criteria as content-oriented anchor points, giving raters an expert-validated reference to assess LLM responses across all evaluated dimensions. These were initially drafted by the first author (SE) based on established radiology resources (Core Radiology [26], Radiopaedia.org [27], Radiology Assistant [28]) and were subsequently independently and blindly reviewed by 2 experienced radiologists (TP and JN) with over 15 and 8 years of clinical and teaching experience, respectively; any discrepancies were resolved through consensus meetings. Subsequently, all LLM responses were also blind-scored independently by evaluating residents.

**Figure 2.**
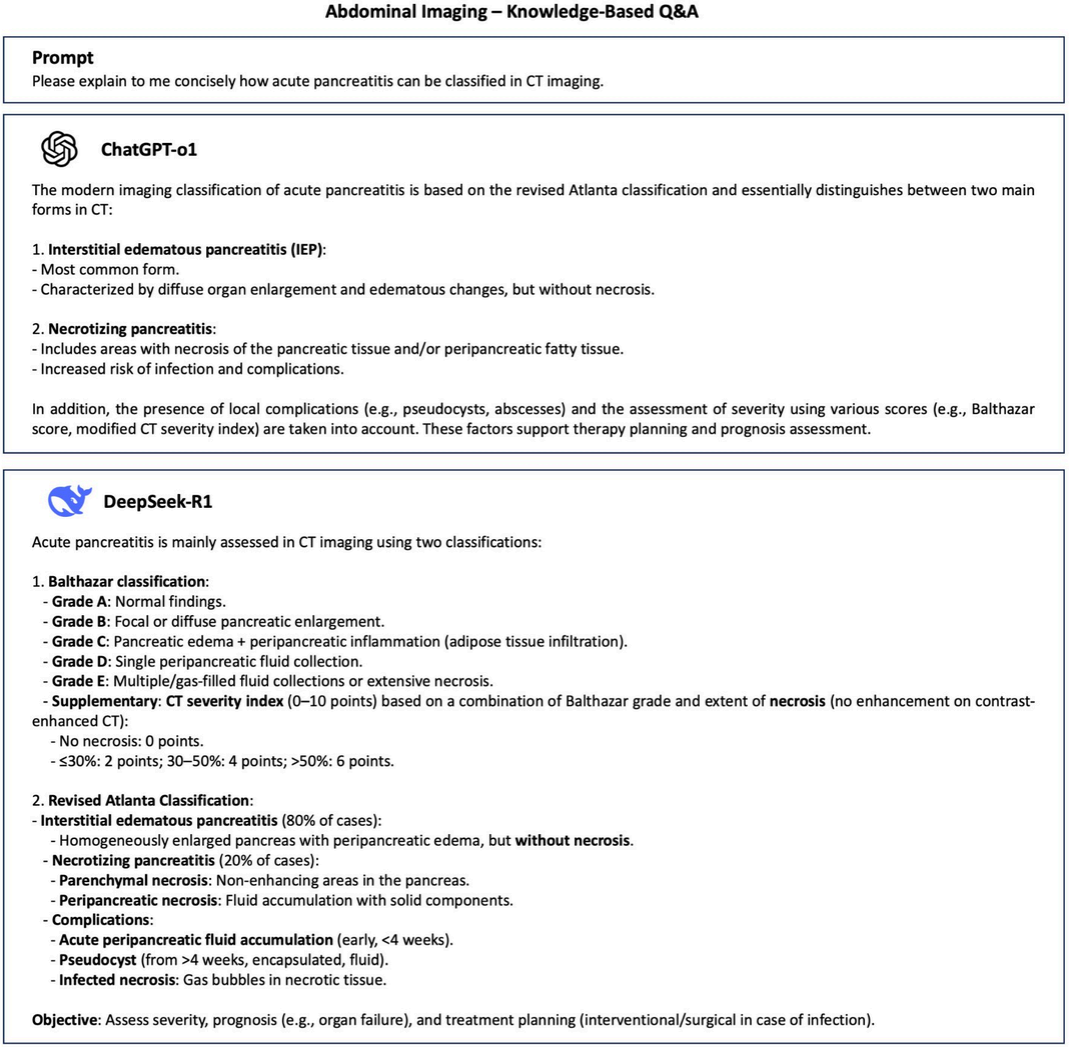
Example of a response for classifying acute pancreatitis on computed tomography imaging. The displayed prompt and model responses have been translated from German to English for publication purposes. CT: computed tomography.

Figure 2 displays exemplary responses from ChatGPT-o1 and DeepSeek-R1 for the classification of acute pancreatitis on computed tomography (CT) imaging.

### Statistical Analysis

#### Paired Design and LLM Comparison (ChatGPT-o1 vs DeepSeek-R1)

The study used a repeated-measures (paired) design. Since each rater evaluated both LLMs on the same set of 27 questions, every question received 2 independent ratings (one per LLM) from the same 7 raters. The 7 ratings were aggregated for each question× criteria combination into a single representative value per LLM by calculating the mean score. Paired differences between the 2 LLMs were tested with a 2-sided Wilcoxon signed-rank test. Holm correction was applied to adjust the significance level for multiple comparisons. As a sensitivity analysis addressing the potential loss of interrater variance through score aggregation, we fitted linear mixed-effects models for each criterion, dimension, and overall score, retaining all individual text-based ratings (n=3402) without aggregation (Table S1 in Multimedia Appendix 3). Each model specified LLM as a fixed effect with crossed random intercepts for rater and question, estimated via restricted maximum likelihood, thereby explicitly accounting for systematic differences in rater severity and question difficulty. Holm correction was applied across the 9 per-criterion tests.

#### Subspecialty-Level Performance

With only 3 question pairs per subspecialty, we did not perform significance testing; instead, subspecialty-level analyses were predefined as exploratory, and we report mean (SD) differences descriptively. With 3 question pairs per subspecialty, these findings are preliminary and subject to variance; they should not be interpreted as generalizable conclusions about model performance across individual radiological domains. This exploratory analysis is best understood as hypothesis-generating.

#### Rating Differences by Training Level (Junior vs Senior Residents)

To examine whether rating behavior differed by training stage, raters were stratified into junior (postgraduate year [PGY] 2, n=4) and senior residents (PGY 3‐5, n=3). We pooled both models’ responses by group and averaged ratings for each criterion, dimension, and overall. Group comparison was performed via Mann-Whitney *U* tests followed by Holm correction. Additionally, junior-senior comparisons were performed for each model.

#### Analysis of Image-Based Question Ratings

The 6 image-based questions were presented only to ChatGPT-o1 due to its ability to process visual inputs; therefore, no direct comparison with DeepSeek-R1 was possible. Given the small sample size of 6 image-based questions, the Mann-Whitney *U* test was applied exclusively across all criteria to compare ChatGPT-o1’s mean text and image ratings, using Holm correction.

#### Interrater Reliability Analysis (ICC)

Interrater agreement was quantified with 2 intraclass correlation coefficients (ICC) computed from a 2-way random-effects model. ICC(2,1) reflects the reliability of a single resident’s rating, whereas ICC(2,k) (k=7) captures the reliability of the mean score averaged across all residents.

#### Statistical Software and Tools

All analyses were conducted in Python 3.9.13 (Pandas 2.2.2, NumPy 1.23.1, SciPy 1.13.1, pingouin 0.5.5, statsmodels 0.14.4, Matplotlib 3.9.2, Seaborn 0.13.2).

## Results

### Analysis of Paired Design and LLM Comparison (ChatGPT-o1 vs DeepSeek-R1)

**Figure 3.**
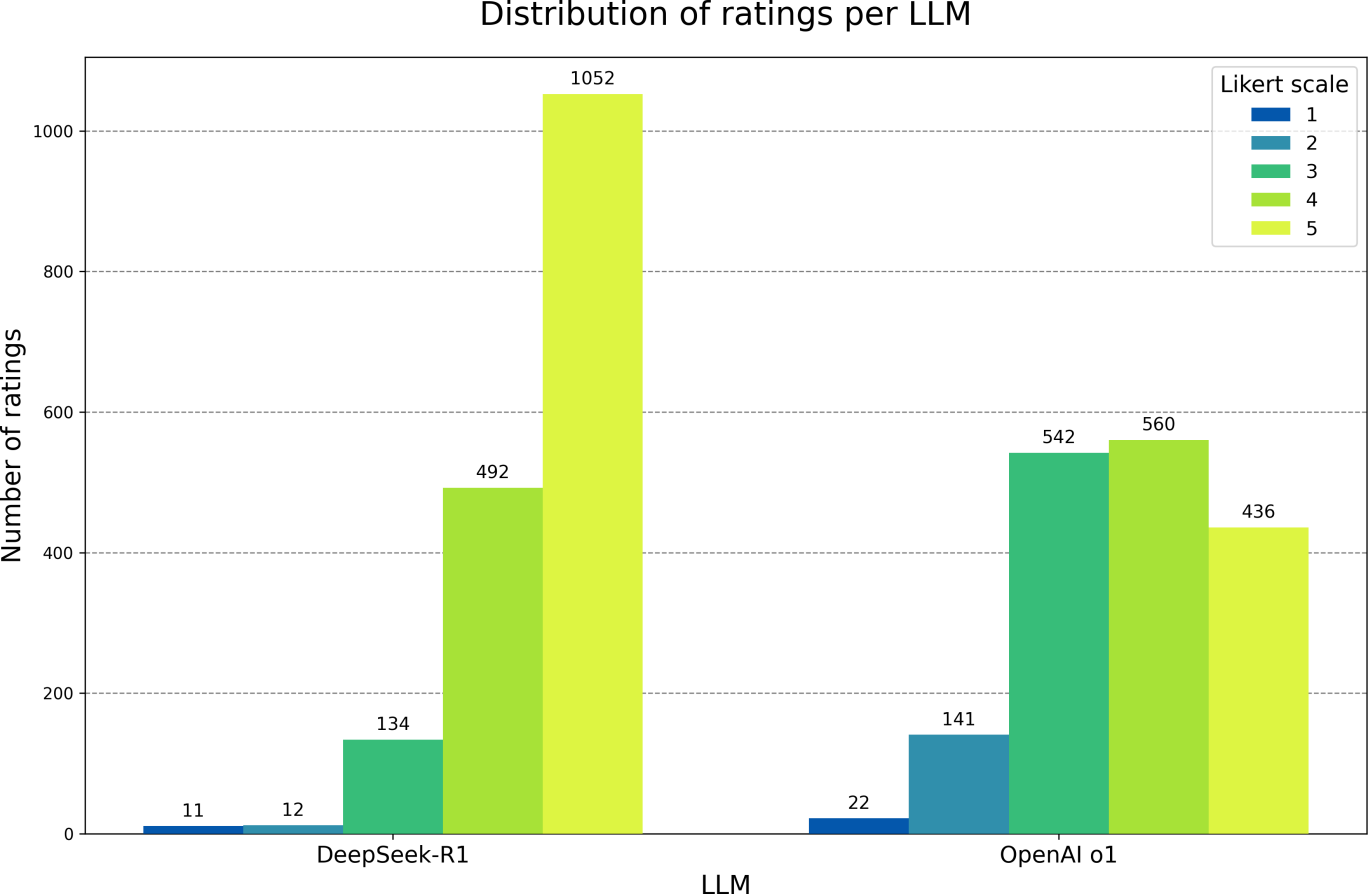
The bar chart displays the distribution of cumulative ratings assigned to 2 large language models, DeepSeek-R1 and ChatGPT-o1, based on evaluations for all 27 text-based questions across nine criteria by all 7 raters. The corresponding absolute rating counts are provided in Table S2 (a) in Multimedia Appendix 3. LLM: large language model.

DeepSeek-R1 consistently outperformed ChatGPT-o1 across all evaluated questions and all 3 rating dimensions and their 9 underlying rating criteria (mean ratings: DeepSeek-R1 4.51, SD 0.73 vs ChatGPT-o1 3.73, SD 0.98; *P*<.001). The difference across all criteria was highly significant (*P*<.001) (Table 1, Figures3  4).

**Table 1.** Statistical comparison between DeepSeek-R1 and ChatGPT-o1 across all evaluation criteria for text-based questions. Mean (SD) values are derived from individual raw ratings (n=189 per criterion, reflecting 7 raters × 27 questions; n=3 overarching rating dimensions). Wilcoxon signed-rank tests were conducted on aggregated mean scores per question pair (n=27), as specified in the methods (statistical analysis).

Rating criteria	DeepSeek-R1	ChatGPT-o1	Wilcoxon paired test (Holm corrected)
	Mean (SD)	Mean (SD)	*P* value
Overall (all criteria)	4.51 (0.73)	3.73 (0.98)	<.001
Factual accuracy	4.55 (0.80)	3.93 (1.02)	<.001
Correctness	4.51 (0.88)	4.19 (1.02)	<.001
Completeness	4.52 (0.84)	3.85 (1.06)	<.001
Precision	4.60 (0.65)	3.74 (0.91)	<.001
Clinical practicality	4.44 (0.71)	3.84 (0.88)	<.001
Comprehensibility	4.63 (0.57)	4.00 (0.77)	<.001
Clinical usefulness	4.41 (0.76)	3.70 (0.94)	<.001
Trustworthiness	4.26 (0.73)	3.81 (0.87)	<.001
Didactic value	4.54 (0.66)	3.43 (0.96)	<.001
Explanation depth	4.59 (0.62)	3.15 (0.81)	<.001
Structure	4.59 (0.62)	3.79 (0.98)	<.001
Learning facilitation	4.42 (0.74)	3.37 (0.95)	<.001

**Figure 4.**
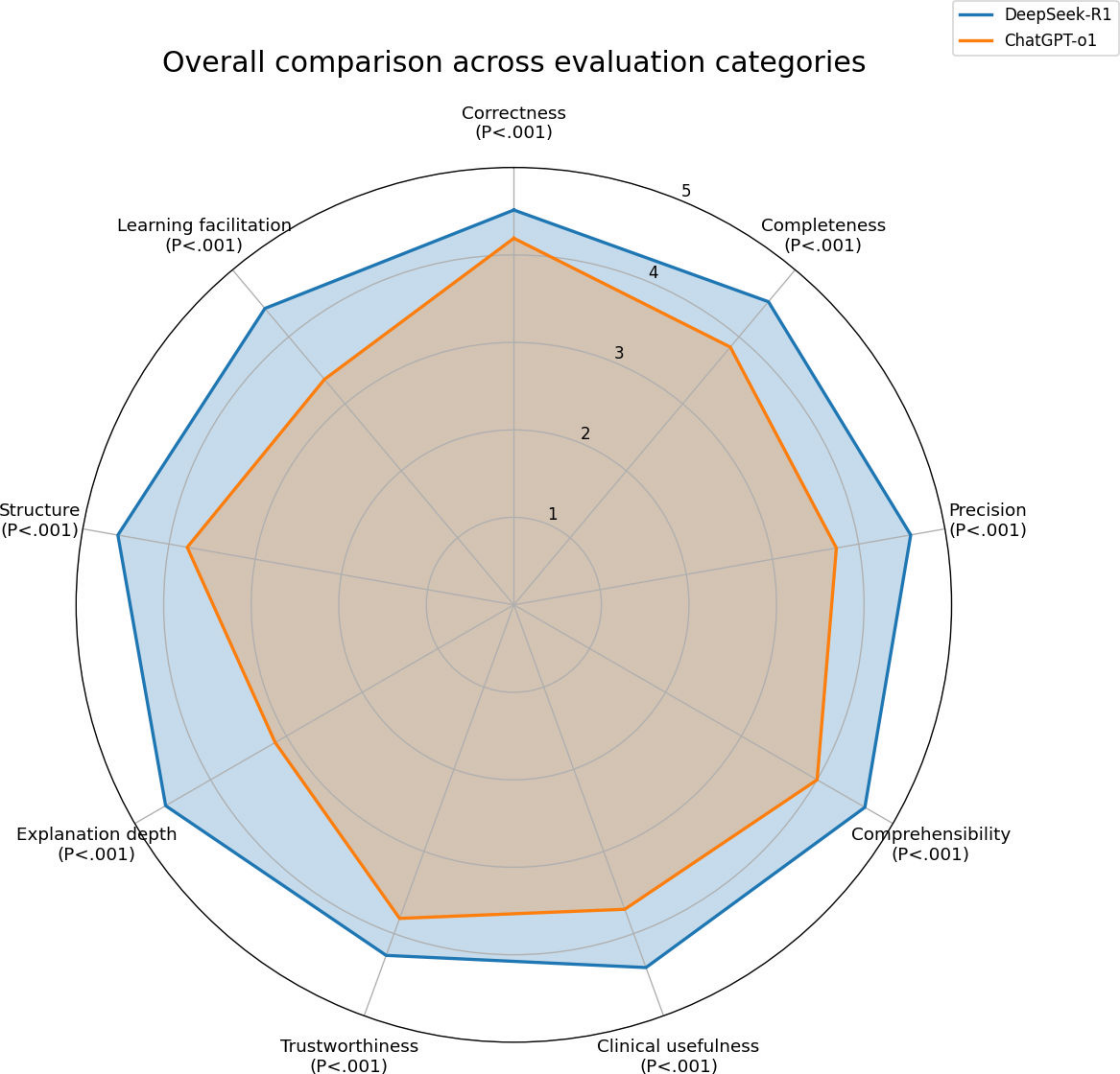
Radar plot comparing mean scores across evaluation criteria for DeepSeek-R1 and ChatGPT-o1 based on evaluations for all 27 text-based questions across 9 criteria by all 7 raters.

**Figure 5.**
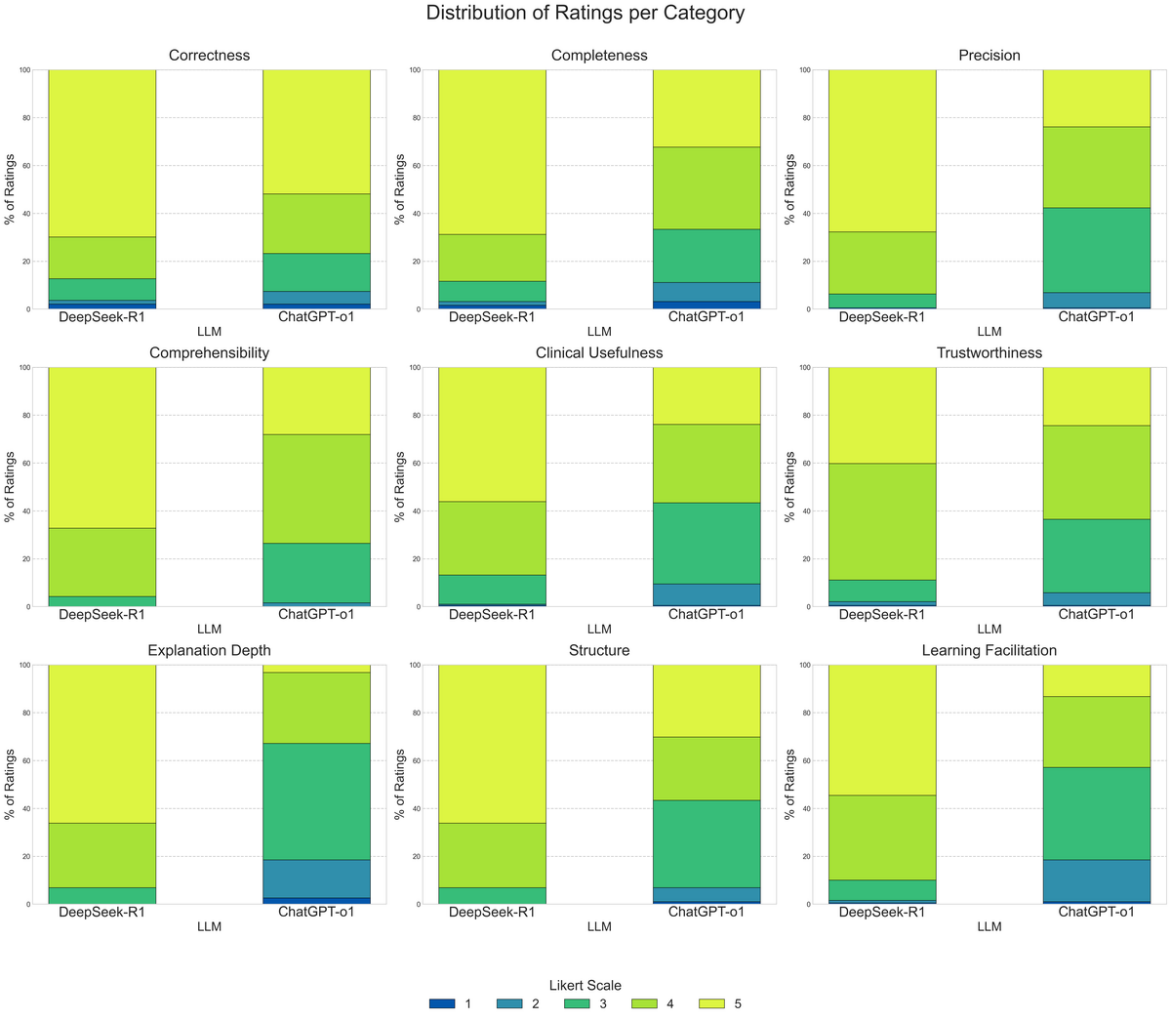
Stacked barplots show the distribution of Likert ratings for all rated criteria DeepSeek-R1 and ChatGPT-o1 for text-based questions. For example, for correctness (n=189 ratings per model), DeepSeek-R1 received mostly top ratings (5: 69.8%, 4: 17.5%), while ChatGPT-o1 showed a broader distribution with fewer top scores (5: 51.9%, 4: 24.9%) and more mid- to low ratings. LLM: large language model.

Overall, ChatGPT-o1 accumulated far more “2” ratings than DeepSeek-R1 (n1=141 vs n2=12) and double the “1” ratings (n1=22 vs n2=11). Cumulative absolute ratings are provided in Figure 3 and relative ratings per criterion in Figure 5.

Across both LLMs combined, ratings of “1” occurred most often for completeness (overall n=9; n1=3 for DeepSeek-R1 vs n2=6 for ChatGPT-o1); ratings of “2” were concentrated in the didactic domain, particularly for learning facilitation (overall n=35; n1=2 for DeepSeek-R1 vs n2=33 for ChatGPT-o1) and explanation depth (overall n=30; n1=0 for DeepSeek-R1 vs n2=30 for ChatGPT-o1). In contrast, ratings of “5” were mostly given to correctness (overall n=230; n1=132 for DeepSeek-R1 vs n2=98 for ChatGPT-o1). Full frequency distributions are reported in Tables S2 (a)-(d) and S3 (a)-(i) in Multimedia Appendix 3. The mixed-effects sensitivity analysis confirmed all primary findings. DeepSeek-R1 scored significantly higher than ChatGPT-o1 across all 9 criteria (all Holm-corrected *P*<.001), with the largest effects observed for explanation depth (β=−1.44) and learning facilitation (β=−1.06). Rater-level and question-level variance were modest relative to residual variance. All 9 significance conclusions were concordant with the aggregated Wilcoxon approach (Table S1 in Multimedia Appendix 3).

### Exploratory Analysis of Subspecialty-Level Performance

**Figure 6.**
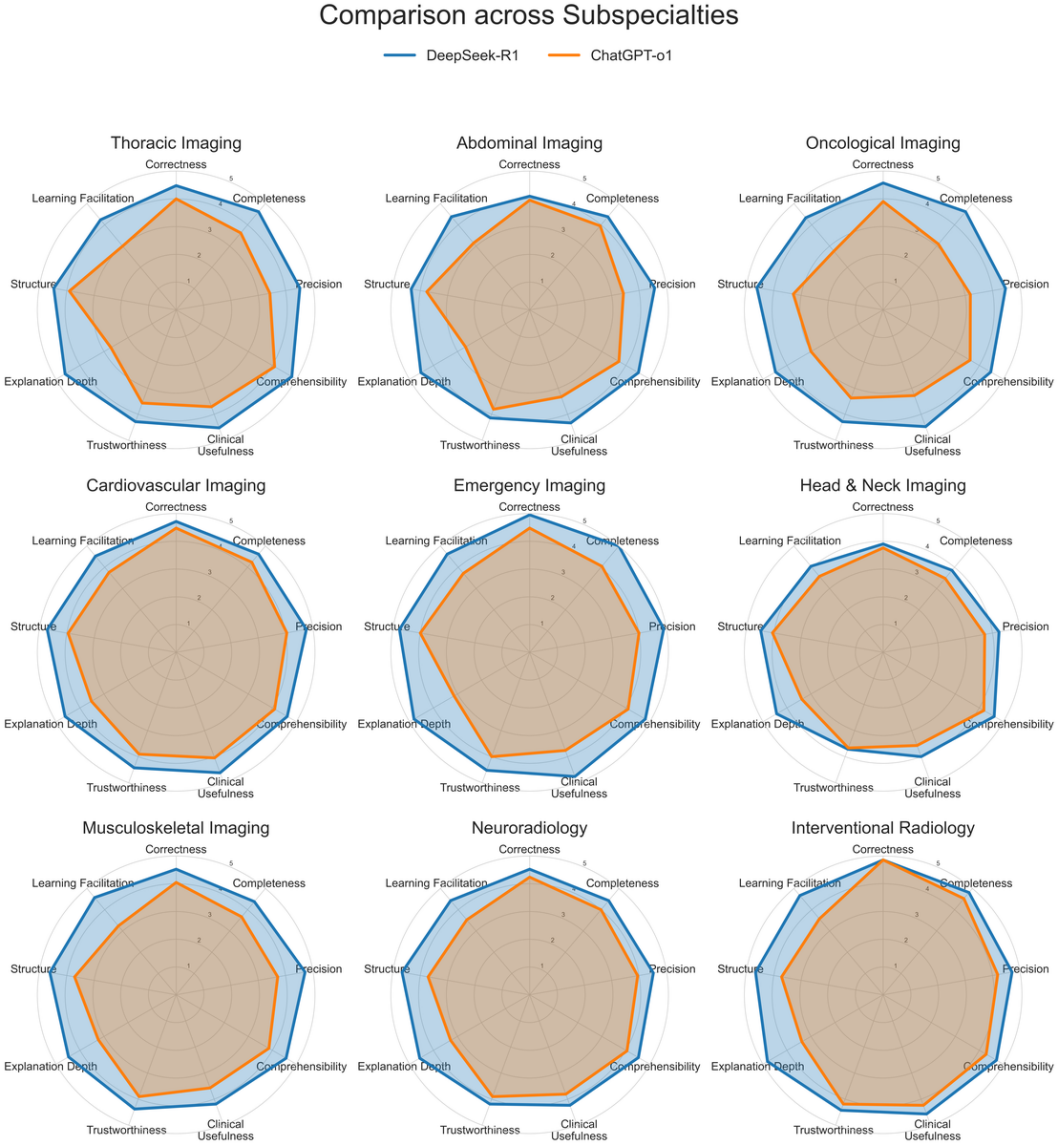
Radar plot panel comparing mean values for DeepSeek-R1 and ChatGPT-o1 across 9 radiology subspecialties by all 7 raters.

In this exploratory subspecialty-level analysis, DeepSeek-R1 consistently descriptively outperformed ChatGPT-o1 across all 9 imaging subspecialties (Table 2) with only a few similar scored criteria for both models, for example, for correctness rating in interventional radiology (Figure 6). With only 3 question pairs per subspecialty, this exploratory analysis should be understood as hypothesis-generating only.

**Table 2.** Overall ratings by imaging subspecialty for DeepSeek-R1 and ChatGPT-o1 (n=9 rating criteria, n=3 questions per subspecialty, n=7 raters).

Subspecialty	DeepSeek-R1, mean (SD)	ChatGPT-o1, mean (SD)
Thoracic imaging	4.54 (0.60)	3.60 (0.89)
Abdominal imaging	4.37 (0.80)	3.53 (0.95)
Oncological imaging	4.48 (0.73)	3.28 (0.99)
Cardiovascular imaging	4.62 (0.59)	4.01 (0.82)
Emergency imaging	4.79 (0.46)	3.92 (0.97)
Head and neck imaging	4.14 (1.13)	3.71 (1.08)
Musculoskeletal imaging	4.49 (0.67)	3.66 (1.06)
Neuroradiology	4.46 (0.66)	3.83 (0.89)
Interventional radiology	4.69 (0.47)	4.11 (0.80)

### Analysis of Rating Differences by Training Level (Junior vs Senior Residents)

No statistically significant differences were observed between junior and senior residents when pooling ratings across both LLMs for any of the 9 rating criteria (Table 3 and Figure S1 in Multimedia Appendix 3).

**Table 3.** Comparison of junior and senior resident ratings for both DeepSeek-R1 and ChatGPT-o1. Parameters are accumulated across all evaluation criteria, including Holm-corrected Mann-Whitney *U* test results.

Rating criteria	Junior residents (n=4)	Senior residents (n=3)	Mann-Whitney-*U* test with Holm correction
	Mean (SD)	Mean (SD)	*P* value
Overall (all criteria)	4.15 (0.65)	4.08 (0.71)	.47
Factual accuracy	4.09 (0.65)	3.98 (0.69)	.47
Correctness	4.21 (0.61)	4.12 (0.69)	≥.99
Completeness	3.93 (0.69)	3.85 (0.73)	≥.99
Precision	4.13 (0.62)	3.96 (0.64)	.73
Clinical practicality	4.11 (0.70)	4.05 (0.82)	.79
Comprehensibility	3.90 (0.79)	3.83 (0.91)	≥.99
Clinical usefulness	4.18 (0.74)	4.20 (0.80)	≥.99
Trustworthiness	4.25 (0.50)	4.11 (0.70)	≥.99
Didactic value	4.26 (0.60)	4.21 (0.59)	.68
Explanation depth	4.31 (0.75)	4.41 (0.63)	≥.99
Structure	4.13 (0.50)	3.91 (0.58)	.32
Learning facilitation	4.33 (0.48)	4.30 (0.44)	≥.99

However, junior residents assigned descriptively higher scores in 7 out of 9 criteria, including precision (juniors 4.13, SD 0.62 vs seniors 3.96, SD 0.64; *P*=.73), trustworthiness (juniors 4.25, SD 0.50 vs seniors 4.11, SD 0.70; *P*≥.99), and structure (juniors 4.13, SD 0.50 vs seniors 3.91, SD 0.58; *P*=.32), although none of these differences were statistically significant. DeepSeek-R1 displayed no subgroup effects (Table S4 in Multimedia Appendix 3), while juniors rated ChatGPT-o1’s overall performance (juniors 3.81, SD 0.64 vs seniors 3.63, SD 0.65; *P*=.02) and structure (juniors 4, SD 0.49 vs seniors 3.57, SD 0.51; *P*=.04) significantly higher (Table S5 in Multimedia Appendix 3).

### Analysis of Image-Based Question Ratings

**Figure 7.**
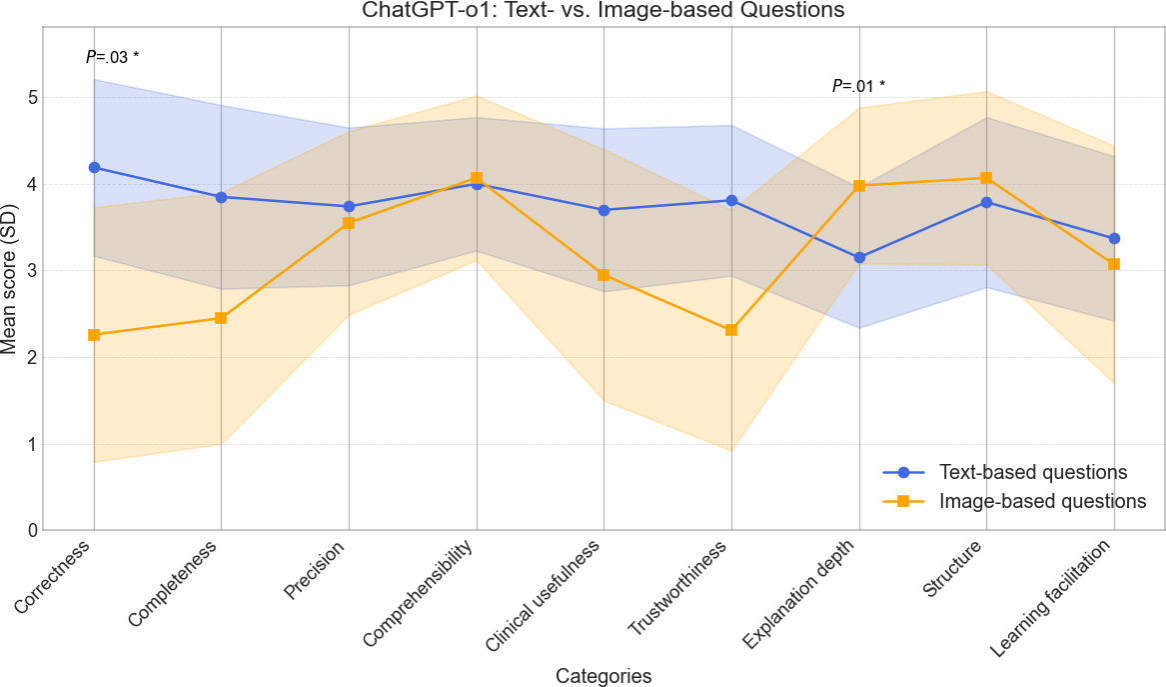
Line plot comparing the mean ratings of the ChatGPT-o1 model for text-based versus image-based questions across every evaluation criterion.

For ChatGPT-o1, image-based questions were rated markedly lower than text-based overall (3.19, SD 1.42 vs 3.73, SD 0.98; *P*=.007), driven by a significant drop in factual accuracy (*P*<.001), especially correctness (*P*=.03), and in clinical practicality (*P*=.03), whereas didactic value got slightly higher rated for the image cases (*P*=.04), mostly due to higher scores in explanation depth (*P*=.01) (Table 4, Figure 7).

Low ratings (1–2) accounted for 34.9% of image-based questions compared to 9.6% for text-based inputs (Table S6 in Multimedia Appendix 3).

**Table 4.** Comparison of ChatGPT-o1 ratings for text- versus image-based questions. Parameters are accumulated across all evaluation criteria using Holm-corrected Mann-Whitney *U* tests.

ChatGPT-o1	Text-based questions (n=27)	Image-based questions (n=6)	Mann-Whitney-*U* test (Holm corrected)
Rating criteria	Mean (SD)	Mean (SD)	*P* value
Overall (all criteria)	3.73 (0.98)	3.19 (1.42)	.007
Factual accuracy	3.93 (1.02)	2.75 (1.45)	<.001
Correctness	4.19 (1.02)	2.26 (1.47)	.03
Completeness	3.85 (1.06)	2.45 (1.45)	.15
Precision	3.74 (0.91)	3.55 (1.06)	.97
Clinical practicality	3.84 (0.88)	3.11 (1.47)	.03
Comprehensibility	4 (0.77)	4.07 (0.95)	.97
Clinical usefulness	3.70 (0.94)	2.95 (1.45)	.34
Trustworthiness	3.81 (0.87)	2.31 (1.39)	.07
Didactic value	3.43 (0.96)	3.71 (1.19)	.04
Explanation depth	3.15 (0.81)	3.98 (0.90)	.01
Structure	3.79 (0.98)	4.07 (1)	.92
Learning facilitation	3.37 (0.95)	3.07 (1.37)	.97

### Interrater Reliability Analysis (ICC)

Single-rater agreement varied across criteria, with ICC(2) ranging from 0.216 (comprehensibility) to 0.608 (factual accuracy), reflecting fair-to-moderate reliability at the individual rater level. Mean rater reliability was consistently strong across all criteria (ICC(2,k)=0.659‐0.916) with an overall ICC(2,k) of 0.89 across all 9 criteria (Table S7 in Multimedia Appendix 3).

## Discussion

### Principal Findings

This study investigated the on-demand diagnostic performance as well as the educational use of 2 state-of-the-art reasoning LLMs, namely ChatGPT-o1 and DeepSeek-R1, across clinically relevant radiology questions. The results demonstrated that DeepSeek-R1 consistently and significantly outperformed ChatGPT-o1 across all evaluated domains, including factual accuracy, clinical practicality, and didactic value. Performance differences were particularly pronounced for didactic criteria such as explanation depth and learning facilitation. Subgroup analyses revealed no major effects of resident training level, indicating generalizable usability across experience levels. Overall, these findings suggest that reasoning LLMs may serve as useful tools for answering radiology-related clinical questions and supporting resident learning in simulated scenarios.

### Performance for Text-Based Questions

While prior studies have examined LLMs for radiology resident training in isolation [29] or for other medical specialties and use-cases (eg, diagnostic reasoning or guideline-based classification tasks) [30-32], to our knowledge, this study displays the first comprehensive comparison within radiology education. Prior findings have been heterogeneous: DeepSeek-R1 outperformed ChatGPT-o1 and Llama 3.1-405B in step-wise diagnostic-reasoning explanations, whereas ChatGPT-o1 excelled at United States Medical Licensing Examination-style exams and radiology report summarization [30]. Both models performed similarly on text-based clinical cases and CT report–based Response Evaluation Criteria In Solid Tumors (RECIST) classifications [30]. The performance gap in our study may be partially explained by different training strategies. While ChatGPT-o1 design and training remain largely undisclosed, DeepSeek-R1 uses supervised fine-tuning, reinforcement learning, and iterative refinement [14]. This approach may support more structured reasoning and improve factual accuracy, which are important for addressing complex, domain-specific tasks in radiology. Furthermore, although GPT-o1 scores slightly higher on the general-purpose massive multitask language understanding-pro benchmark [15], our findings suggest that such metrics do not reliably predict performance in highly specialized contexts such as radiology resident education.

ChatGPT-o1 got nearly twice as many “1” and over 10 times more “2” ratings as DeepSeek-R1, mainly for completeness, learning facilitation, and explanation depth. Most “5” ratings were awarded for correctness, indicating that both models can be accurate but differ markedly in depth and educational value. Rare yet serious gaps in completeness can threaten patient safety, while didactic shortcomings may reduce learning impact. Ten of the eleven “1” ratings for DeepSeek-R1 originate from a single head-and-neck question describing a T2-hyperintense, well-defined, non-enhancing lesion at the mandibular angle. Both models incorrectly assumed osseous origin, failing to recognize the imaging pattern as consistent with a branchial cleft cyst. While more explicit phrasing specifying a soft-tissue lesion could have guided the models toward the correct diagnosis, the combination of MRI characteristics described represents a recognizable radiological pattern that a clinically experienced reader would be expected to correctly identify. The shared misinterpretation therefore reflects both the sensitivity of LLM outputs to prompt phrasing and a genuine limitation in contextual clinical reasoning, highlighting the importance of precise prompting in clinical and educational applications. Although DeepSeek-R1 outperformed ChatGPT-o1, it still produced eleven “insufficient” responses, underscoring the need for mandatory human verification. Infrequent but dangerous errors outweigh minor flaws, demanding layered safeguards such as automated uncertainty detection, structured review checklists, and human approval for high-risk cases.

**Figure 8.**
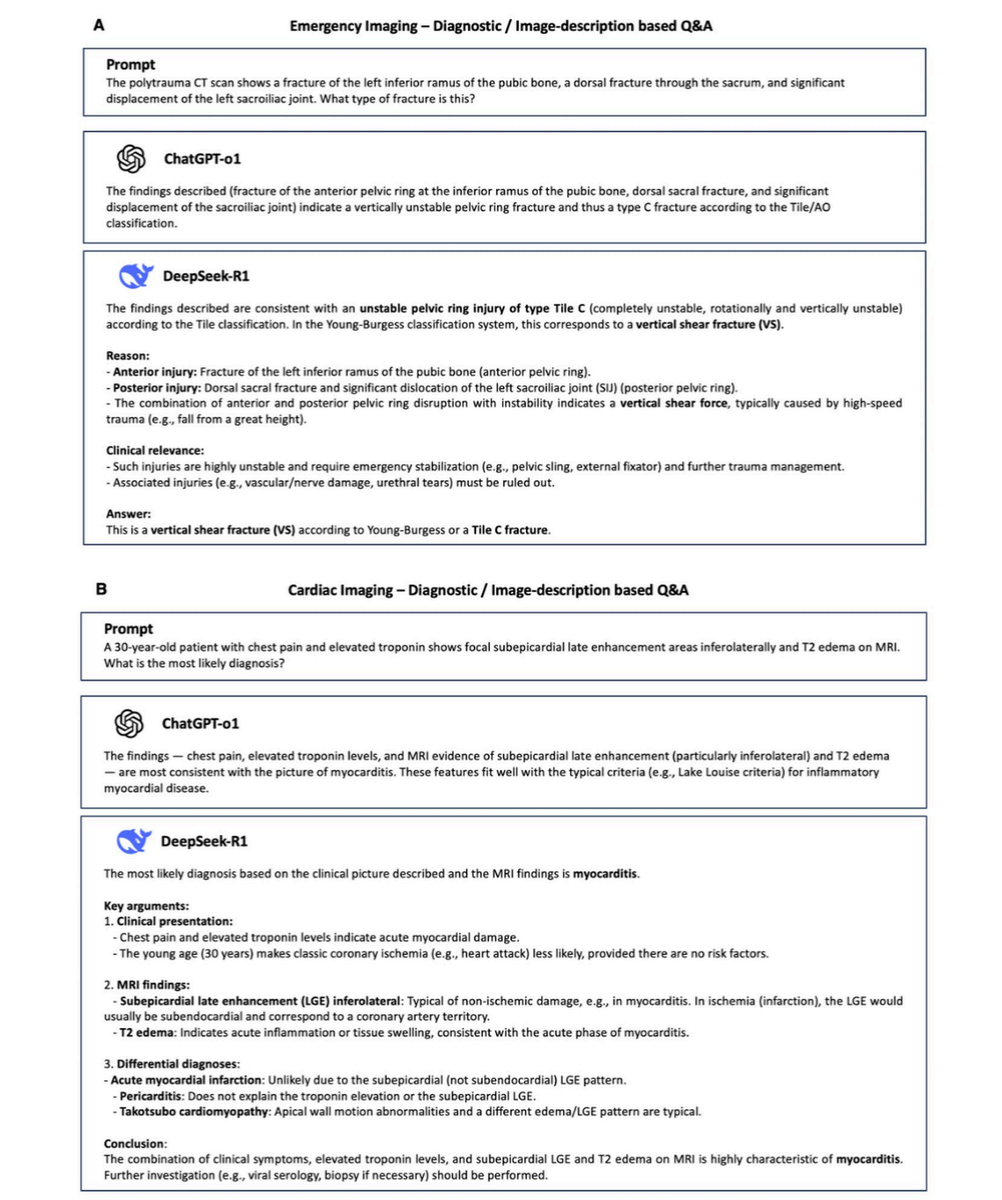
Representative model outputs from emergency (A) and cardiac (B) imaging prompts illustrating differences in explanation depth, reasoning structure, and didactic clarity between ChatGPT-o1 and DeepSeek-R1. The displayed prompts and model responses have been translated from German to English for publication purposes. CT: computed tomography.

ChatGPT-o1 received a “1” rating for providing insufficient detail (50 words vs DeepSeek-R1’s 157 words). While previous studies have noted DeepSeek’s tendency toward lengthy, repetitive responses [16,30], our findings demonstrate that DeepSeek-R1 received its highest rating advantage in explanation depth, reflecting that reviewers found its more detailed answers especially helpful for learning. While residents may favor concise, context-aware explanations for rapid comprehension, such compactness can hinder deeper learning by overlooking essential details and detailed reasoning required to understand complex radiological scenarios. To illustrate these differences in didactic value, 2 representative model outputs from emergency and cardiac imaging are displayed in Figure 8.

A potential verbosity bias may have specifically affected the didactic value ratings. Given that DeepSeek-R1 consistently produced longer responses and simultaneously received its greatest rating advantage in explanation depth and learning facilitation, criteria that are inherently susceptible to length-related perception effects, the possibility that raters equated response length with educational quality cannot be excluded. Future evaluations could explore structured rating frameworks that explicitly separate content quality from response length to mitigate this effect. Matching superiority across all questions, DeepSeek-R1 also appears to outperform ChatGPT-o1 in all 9 imaging subspecialties, with minor overlaps (eg, correctness in interventional radiology), likely due to a low number of questions per subspecialty (n=3). As noted in the “Limitations” section, the subspecialty analysis lacks statistical power for domain-specific conclusions. We used standardized single-turn, one-shot batch prompting to enable a strictly paired model comparison and avoid rater-dependent variability, which may render our findings a conservative estimate of real-world performance, where iterative re-prompting and answer refinement can further improve output quality.

### ChatGPT-o1’s Performance for Image-Based Questions

ChatGPT-o1’s accuracy dropped on image-based cases driven by declines in factual accuracy and clinical practicality, whereas its didactic scores rose slightly. ChatGPT-o1 failed to identify straightforward MRI findings, for example, mislabeling a lumbar disc herniation as spondylodiscitis and a meniscal tear as a Baker’s cyst, echoing studies that GPT-4 handles text-only radiology questions far more accurately than those requiring image interpretation [33,34]. This underscores that current large language models excel on text but struggle with visuals, likely due to limited paired image-text supervision and the complexity of visual signs. Several factors may have contributed to ChatGPT-o1’s decreasing performance on image-based cases. First, our prompts were relatively generic and did not explicitly include specific image descriptions (eg, attenuation and enhancement) or clinical context (eg, patient history, laboratory values, or prior imaging), reducing the model’s differential generation reliability. Second, the mismatch between the model’s predominantly text-based and generic vision-language pretraining and our specialized radiologic cases (eg, high-field MRI spine sequences) may have impaired its ability to recognize expected imaging features. Third, limitations in image resolution and potential compression artifacts may have obscured subtle findings.

### Rating Differences by Training Level (Junior vs Senior Residents)

Junior residents were awarded higher scores on 7 of 9 criteria (exceptions were clinical usefulness and explanation depth), yet overall ratings did not differ significantly from seniors. This may reflect (1) a clear, structured scoring rubric that guided consistent ratings across experience levels or (2) difficulties in detecting subtle differences in answer quality, something future studies could address through rater training or reference standards. Interestingly, junior residents rated ChatGPT-o1 significantly higher than seniors in both overall score and structure. This was not observed for DeepSeek-R1, possibly reflecting its more consistent, higher-quality output. In contrast, ChatGPT-o1’s more variable style may have appeared more pedagogically accessible to juniors, while advanced residents judged it more critically. These findings do not imply equivalent usability across experience levels. Differences in scoring may instead reflect variation in comfort with artificial intelligence tools, especially LLMs, as well as differing expectations for detail and explanation depth, or greater familiarity of senior residents with typical diagnostic pitfalls and subtle error patterns. Further research with larger and more heterogeneous resident groups will be required to understand how LLM performance is perceived across stages of training. Single-rater reliability was moderate (ICC≈0.6), rising to near-excellent across 7 raters (ICC≈0.89) [35]. In this study, our evaluation primarily reflects how trainees perceive LLM outputs, and future work should also involve attending radiologists to better characterize staff-level expectations and critical appraisal of model performance.

### Open-Source vs Proprietary Models

Open-source models such as DeepSeek-R1 prioritize transparency, local deployment, and custom fine-tuning, benefiting health care research and data-sensitive clinical applications, though they require greater technical resources and expertise. DeepSeek-R1’s openly released weights and low resource requirements may allow large health care organizations to run the model locally for in-house fine-tuning and deployment. However, the limited information about its training data and the absence of training code raise open questions around data provenance, reproducibility, and responsible model governance [16].

### Responsible Deployment for Global Health Care Equity

Our findings underscore the need to examine the broader implications of open-source LLM deployment for global health‐care equity. Although open‐source, locally deployed LLMs could reduce costs and support under-resourced regions, disparities in high-performance computing and high-quality data risk widening the digital divide [36]. Developed regions may refine these technologies more rapidly, widening the gap with resource-constrained areas. Future research should extend beyond technical model benchmarking to explore open-source collaboration, local deployment, and low-cost optimizations that ensure equitable global health care delivery.

Additionally, policy measures, international cooperation, and resource-sharing initiatives must be explored to prevent monopolies and ensure that LLMs strengthen, rather than exacerbate, global health care and educational equity.

### Future Directions

Future research should explore how multimodal LLMs, capable of processing radiological images and accompanying text, could support more complex, realistic training scenarios. In addition, prospective studies comparing and combining LLM-assisted learning with conventional teaching methods are needed to assess long-term educational impact, for example through structured training modules with pre- and post-knowledge evaluations, longitudinal follow-up of resident performance, and objective structured clinical examinations incorporating LLM-supported components. LLMs could support resident training through real-time case questions and answers, automated learn card generation, dynamic case simulations (combining patient history and imaging), guided reporting practice and quality assurance (“second-reads”), and on-demand guideline summaries or quiz creation to keep residents up to date. Further work should optimize prompt strategies, evaluate curriculum integration, and assess user trust, error types, and practical safeguards for clinical use.

### Limitations

This study has several limitations. First, the rating scale, while multidimensional, remains subjective and is based on assessments from 7 residents at a single institution, which limits external validity. Since model outputs were evaluated solely by radiology residents (PGY 2‐5), subtle factual errors or hallucinations more readily identified by experienced attending radiologists may have gone undetected, potentially leading to an overestimation of factual accuracy and overly optimistic safety assessments. Future studies should include attending-level evaluation to establish a more rigorous and safety-relevant accuracy benchmark. Expert input came from one senior general radiologist and one neuroradiologist rather than a formal subspecialty panel.

Second, the small question set (27 text-based and 6 image-based) constrains generalizability, though it was kept intentionally compact given the extensive multicriteria evaluation required per response. With 3 question pairs per subspecialty, findings at this level are preliminary and should not be interpreted as generalizable conclusions across individual radiological domains. Additionally, 1 question included an explicit conciseness instruction that may have been in tension with depth-sensitive evaluation criteria, potentially disadvantaging the model ChatGPT-o1 that adhered more closely to that constraint. Future work should expand the question set and involve multi-institutional and subspecialty expert input.

Third, blinding integrity may have been partially compromised, as the consistently greater length and detail of DeepSeek-R1 responses compared to ChatGPT-o1 may have allowed raters to infer model identity from stylistic characteristics alone. Should raters have perceived longer responses as more comprehensive, this could have introduced a systematic bias in favor of DeepSeek-R1, potentially contributing to the observed performance gap between the 2 models.

Fourth, image-based questions were sourced from Radiopaedia.org, a publicly accessible platform potentially indexed during model pretraining, introducing a data contamination risk for the image-based results. Similarly, while text-based questions were derived from clinical experience, the underlying declarative medical knowledge was likely present in the models’ pretraining corpora.

Fifth, the comparison of ChatGPT-o1’s text-based and image-based performance is subject to a notable confound: the 2 conditions involve different clinical cases, and lower image-based scores may in part reflect the specific difficulty of those 6 cases in addition to potential visual processing limitations. A matched design presenting identical cases as both image inputs and text descriptions would be required to fully isolate modality effects.

Sixth, as all questions were submitted in German, observed performance differences may partly reflect differential multilingual robustness of the 2 models rather than domain-specific reasoning capability alone. While this choice reflects real-world usage conditions for German-speaking residents, generalizability to other language settings remains limited and should be addressed in future studies.

Seventh, the inherently subjective nature of the didactic and clinical practicality criteria limits the extent to which standardized reference answers can ensure consistent scoring, potentially introducing additional inter-rater variability.

Eighth, rapid LLM evolution may limit our findings’ applicability to upcoming model versions.

### Conclusions

This study demonstrates that DeepSeek-R1 significantly outperformed ChatGPT-o1 across all 3 dimensions: factual accuracy, clinical practicality, and didactic value for text-based radiology questions, with highly significant differences across all 9 rating criteria. For ChatGPT-o1, image-based performance was significantly lower than text-based performance, particularly in factual accuracy and clinical practicality. No statistically significant differences were observed between junior and senior resident raters when pooling both models. These findings provide early, controlled evidence that reasoning LLMs can produce clinically and educationally relevant responses to radiology residency questions, while also demonstrating the limitations of current vision-language capabilities. Both models still produced factually insufficient responses in a subset of cases, underscoring the continued necessity of human expert oversight.

## Supplementary material

10.2196/86974 Multimedia Appendix 1 Complete list of questions submitted to DeepSeek-R1 and ChatGPT-o1.

10.2196/86974 Multimedia Appendix 2 Rating criteria definitions and Likert scale anchors.

10.2196/86974 Multimedia Appendix 3 Additional statistical tables and figures.

## Abbreviations

CT: computed tomography
ICC: intraclass correlation coefficient
LLM: large language model
MRI: magnetic resonance imaging
PGY: postgraduate year
RECIST: Response Evaluation Criteria In Solid Tumors
