# Supplementary material for: Supporting Radiology Resident Education and Clinical Decision-Making With Large Language Models: Comparative Study of Reasoning Models DeepSeek-R1 and ChatGPT-o1
Source: JMIR AI. 2026 Jun 26;5:e86974. doi: 10.2196/86974 (PMC13309062; doi:10.2196/86974)
Supplement: Multimedia Appendix 1 [file ai-v5-e86974-s001.docx]

**Supplementary Text 1** Full list of provided questions translated to English

**Text-based questions**

**Thoracic Imaging**
1. What are five typical signs of active tuberculosis on CT?
2. In a patient with respiratory symptoms and diffuse nodular pulmonary seeding including cavitation, what are the three most likely differential diagnoses??
3. A patient without prior disease presents with dyspnea. CT shows bilateral ground-glass opacities with “crazy-paving” and consolidation. What are the three most likely differentials?

**Abdominal Imaging**
4. How is acute pancreatitis classified on CT?
5. A cystic pancreatic lesion with thick enhancing walls is seen on CT. What is the most likely diagnosis and what are three important differentials?
6. CT shows segmental thickening of the terminal ileum with hyperenhancement and fat wrapping. What is the most likely diagnosis and three key differential diagnoses?

**Oncologic Imaging**
7. How does clear cell renal cell carcinoma typically appear on contrast-enhanced CT?
8. A solid hypoechoic thyroid nodule with microcalcifications, taller-than-wide shape, and irregular borders is seen on ultrasound. What is the malignancy risk and which classification system applies?
9. A liver lesion shows peripheral arterial enhancement and homogeneous venous fill-in. What are the three most likely differential diagnoses?

**Cardiovascular Imaging**
10. What is the minimal femoral artery diameter required for transfemoral transcatheter aortic valve implantation (TAVI) access and what other CT-detectable risk factors are relevant?
11. A 70–80% calcified, eccentric left anterior descending artery (LAD) stenosis is seen on CT angiography (CTA) with positive remodeling. How is this graded using Coronary Artery Disease Reporting and Data System (CAD-RADS)?
12. A 30-year-old with chest pain and elevated troponin shows subepicardial late gadolinium enhancement (LGE) inferolaterally and T2 edema in MRI. What is the most likely diagnosis?

**Emergency Imaging**
13. How is splenic injury graded on trauma CT, and what is the significance of contrast extravasation?
14. Trauma CT shows fracture of the inferior pubic ramus, dorsal sacral fracture, and displacement of the left Sacroiliac (SI) joint. What type of pelvic fracture is this?
15. A patient presents with abdominal pain and portal venous gas, but otherwise normal CT findings. What is the most likely diagnosis and what else should be evaluated?

**Head & Neck Imaging**
16. What are three typical CT signs of acute mastoiditis?
17. A T2-hyperintense, well-demarcated lesion at the mandibular angle shows no enhancement. What is the most likely diagnosis and top three differentials?
18. A 60-year-old with chronic symptoms shows a soft tissue mass in the nasal cavity with bony remodeling but no destruction. What is the most likely diagnosis and three differential diagnoses?

**Musculoskeletal Imaging**
19. What are the three most common primary bone tumors in patients aged 10–30 years?
20. MRI shows diffuse bone marrow edema in the medial tibial plateau without fracture or trauma, with load-dependent pain. What is the most likely diagnosis and top three differential diagnoses?
21. A CT shows inhomogeneous bone and adjacent soft tissue defect. How can CT help differentiate between acute and chronic osteomyelitis?

**Neuroradiology**
22. What are the three key MRI features to differentiate glioblastoma from low-grade astrocytoma?
23. A spinal MRI shows a T2-hyperintense lesion across several cervical vertebral levels with homogeneous enhancement and no mass effect. What is the most likely diagnosis and top three differentials?
24. In suspected stroke, non-contrast CT (NCCT) shows subtle hypodensity and CTA confirms M1 occlusion. CT perfusion shows prolonged time-to-peak (TTP) and mean-transit-time (MTT) with preserved cerebral blood volume (CBV) and reduced cerebral blood flow (CBF). How can this be interpreted and what are the reperfusion prospects?

**Interventional Radiology**
25. What are the key indications for transjugular intrahepatic portosystemic shunt (TIPS) placement?
26. Post-cryoablation CT shows a centrally necrotic lesion with surrounding ground-glass halo. How should this be interpreted?
27. Post transarterial chemoembolization (TACE) MRI shows a centrally T1-hyperintense area within a treated hepatocellular carcinoma (HCC) without enhancement. How should this be interpreted?

**Image-based questions (based on CT and MRI images provided in Figure 1)**

1. **Please describe the radiological findings on this non-contrast CT image** (location, density, morphology, etc.). What is the most likely diagnosis? What are three important differential diagnoses to consider?

2. **Please describe the radiological findings on this contrast-enhanced CT image** (location, density, morphology, etc.). What is the most likely diagnosis? What are three important differential diagnoses to consider?

3. **Please describe the radiological findings on this contrast-enhanced CT image** (location, density, morphology, etc.). What is the most likely diagnosis? What are three important differential diagnoses to consider?

4. **Please describe the radiological findings on this MRI image.** What is the most likely diagnosis? What are three important differential diagnoses to consider?

5. **Please describe the radiological findings on this MRI image.** What is the most likely diagnosis? What are three important differential diagnoses to consider?

6. **Please describe the radiological findings on this MRI image.** What is the most likely diagnosis? What are three important differential diagnoses to consider?
