# Supplementary material for: Supporting Radiology Resident Education and Clinical Decision-Making With Large Language Models: Comparative Study of Reasoning Models DeepSeek-R1 and ChatGPT-o1
Source: JMIR AI. 2026 Jun 26;5:e86974. doi: 10.2196/86974 (PMC13309062; doi:10.2196/86974)
Supplement: Multimedia Appendix 2 [file ai-v5-e86974-s002.docx]

**Table S1.** Overview of Evaluation Criteria with Definitions and Anchored Likert Scale Descriptions Used for Radiology Resident Assessment.

| **Criteria** | **Definition** | **Likert-Scale** |
| --- | --- | --- |
| Factual Accuracy | | |
| Correctness | Does the response align with established radiological teaching, guidelines, and validated expert information? | 1 = Incorrect or misleading  2 = Partially correct, contains relevant errors  3 = Generally correct, minor inaccuracies  4 = Largely correct, only marginal deviations  5 = Completely correct |
| Completeness | Are all relevant or most important aspects, diagnoses, or differentials mentioned? | 1 = Key content is missing 2 = Several important points are missing 3 = Central points present, but somewhat incomplete 4 = Nearly complete 5 = Fully comprehensive, all relevant content included |
| Precision | How clear and specific is the formulation of the response? Does it avoid vague phrases or overly general statements? | 1 = Very vague and imprecise 2 = Roughly outlined, unclear formulations 3 = Adequate but not very detailed 4 = Clearly and well formulated 5 = Very precise, detailed, and to the point |
| Clinical Practicality | | |
| Comprehensibility | Is the language appropriate, clear, and understandable for radiology residents? | 1 = Incomprehensible or confusing 2 = Partially difficult to follow 3 = Generally understandable 4 = Clearly understandable with concise language 5 = Exceptionally clear, easily accessible even for learners |
| Clinical Usefulness | Is the response practically applicable and clinically useful? | 1 = Incomprehensible or confusing 2 = Partially difficult to follow 3 = Generally understandable 4 = Clearly understandable with concise language 5 = Exceptionally clear, easily accessible even for learners |
| Trustworthiness | Would one rely on this answer when making a real-life clinical decision? | 1 = No practical relevance 2 = Minimally helpful 3 = Situationally helpful 4 = Useful in many situations 5 = Highly practical and directly applicable in clinical context |
| Didactic Value | | |
| Explanation Depth | Does the response explain underlying relationships or remain superficial? | 1 = No explanation, just listing 2 = Very superficial 3 = Partially explanatory 4 = Well explained with context 5 = Didactically strong, supports deep understanding |
| Structure | Is the response logically structured (e.g., image description → diagnosis → differential diagnosis)? | 1 = Unstructured and confusing 2 = Partially disorganized 3 = Basic structure recognizable 4 = Well-structured 5 = Clearly organized and logically built |
| Learning Facilitation | Does the response contribute to education? Does it include ‘aha’ moments or memorable insights**?** | 1 = No learning effect 2 = Minimally inspiring 3 = Solid educational impulse 4 = Good educational value 5 = Excellent educational value with "aha" effect |
