# Supplementary material for: Supporting Radiology Resident Education and Clinical Decision-Making With Large Language Models: Comparative Study of Reasoning Models DeepSeek-R1 and ChatGPT-o1
Source: JMIR AI. 2026 Jun 26;5:e86974. doi: 10.2196/86974 (PMC13309062; doi:10.2196/86974)
Supplement: Multimedia Appendix 3 [file ai-v5-e86974-s003.docx]

**Table S1.** Mixed-Effects Model Sensitivity Analysis for text-based questions: Per-Criterion Resuls

**Panel A: Per-Criterion Analysis**

| **Criterion** | **Dimension** | **β** | **SE** | **z** | **p** | **p (Holm)** | **95% CI** | **Wilcoxon p (Holm)** | **Concord- ance** |
| --- | --- | --- | --- | --- | --- | --- | --- | --- | --- |
| Correctness | Factual Accuracy | -0.323 | 0.052 | -6.23 | <.001 | <.001 | [-0.42, -0.22] | .0004 | Yes |
| Completeness | Factual Accuracy | -0.677 | 0.069 | -9.75 | <.001 | <.001 | [-0.81, -0.54] | <.001 | Yes |
| Precision | Factual Accuracy | -0.862 | 0.067 | -12.84 | <.001 | <.001 | [-0.99, -0.73] | <.001 | Yes |
| Comprehensibility | Clinical Practicality | -0.630 | 0.056 | -11.31 | <.001 | <.001 | [-0.74, -0.52] | <.001 | Yes |
| Clinical Usefulness | Clinical Practicality | -0.709 | 0.063 | -11.18 | <.001 | <.001 | [-0.83, -0.58] | <.001 | Yes |
| Trustworthiness | Clinical Practicality | -0.450 | 0.056 | -8.05 | <.001 | <.001 | [-0.56, -0.34] | <.001 | Yes |
| Explanation Depth | Didactic Value | -1.444 | 0.070 | -20.76 | <.001 | <.001 | [-1.58, -1.31] | <.001 | Yes |
| Structure | Didactic Value | -0.804 | 0.071 | -11.36 | <.001 | <.001 | [-0.94, -0.67] | <.001 | Yes |
| Learning Facilitation | Didactic Value | -1.058 | 0.070 | -15.11 | <.001 | <.001 | [-1.20, -0.92] | <.001 | Yes |

**Panel B: Per-Dimension Analysis**

| **Dimension** | **β** | **SE** | **z** | **p** | **95% CI** |
| --- | --- | --- | --- | --- | --- |
| Factual Accuracy | -0.621 | 0.038 | -16.24 | <.001 | [-0.696, -0.546] |
| Clinical Practicality | -0.596 | 0.035 | -17.07 | <.001 | [-0.665, -0.528] |
| Didactic Value | -1.102 | 0.040 | -27.59 | <.001 | [-1.181, -1.024] |

**Panel C: Variance Components (Overall Model)**

- Overall fixed effect (all criteria pooled): β = −0.773, SE = 0.023, z = −33.53, p < .001, 95% CI [−0.818, −0.728]
- Rater random intercept variance: σ² = 0.123
- Question random intercept variance: σ² = 0.183
- Residual variance: σ² = 0.452

*Abbreviations*: β, fixed-effect coefficient (negative values indicate higher scores for DeepSeek-R1); SE, standard error; z, z-statistic (Wald test); p, uncorrected p-value; p (Holm), p-value after Holm correction for multiple comparisons; 95% CI, 95% confidence interval of β; Wilcoxon p (Holm), Holm-corrected p-value from the primary aggregated Wilcoxon signed-rank test; Concordance, whether both analytical approaches yield the same significance conclusion at α = .05; σ², variance component; REML, restricted maximum likelihood; LMM, linear mixed-effects model.

Linear mixed-effects models with LLM as fixed effect and rater and question as crossed random intercepts (score ~ LLM + (1|rater) + (1|question)), estimated via REML. Reference category: DeepSeek-R1; negative coefficients indicate higher scores for DeepSeek-R1. Holm correction applied across nine criteria. The rightmost columns show the corresponding Holm-corrected Wilcoxon signed-rank p-values from the primary analysis and whether both approaches yield the same significance conclusion (α = .05). N = 3,402 individual ratings (27 questions × 7 raters × 2 LLMs × 9 criteria).

Concordance: 9/9 criteria yielded identical significance conclusions between the aggregated Wilcoxon signed-rank test and the mixed-effects model, confirming that the primary analysis conclusions are robust to the choice of analytical strategy.

**Table S2. (a)-(d)** Grading of the performance of two Large Language Models (DeepSeek-R1 and ChatGPT-o1) in text-based questions across nine evaluation criteria pooled overall and for their overarching rating dimensions factual accuracy, clinical practicality and didactic value. Values represent the absolute cumulative number of ratings (Likert scores 1–5), shown as count (percentage).

| (a) | **Overall** | |
| --- | --- | --- |
| Rating | DeepSeek-R1 | ChatGPT-o1 |
| 5 | 1052 (61.86%) | 436 (25.64%) |
| 4 | 492 (28.93%) | 560 (32.92%) |
| 3 | 134 (7.88%) | 542 (31.87%) |
| 2 | 12 (0.71%) | 141 (8.29%) |
| 1 | 11 (0.65%) | 22 (1.29%) |
| Mean ± SD | 4.51 ± 0.73 | 3.73 ± 0.98 |

| (b) | **Factual Accuracy** | |
| --- | --- | --- |
| Rating | DeepSeek-R1 | ChatGPT-o1 |
| 5 | 390 (68.79%) | 204 (35.97%) |
| 4 | 119 (20.98%) | 176 (31.04%) |
| 3 | 44 (7.76%) | 139 (24.52%) |
| 2 | 6 (1.06%) | 37 (6.53%) |
| 1 | 8 (1.41%) | 11 (1.94%) |
| Mean ± SD | 4.55 ± 0.80 | 3.93 ± 1.02 |

| (c) | **Clinical Practicality** | |
| --- | --- | --- |
| Rating | DeepSeek-R1 | ChatGPT-o1 |
| 5 | 309 (54.49%) | 144 (25.40%) |
| 4 | 204 (35.97%) | 222 (39.16%) |
| 3 | 48 (8.47%) | 169 (29.80%) |
| 2 | 4 (0.70%) | 30 (5.29%) |
| 1 | 2 (0.35%) | 2 (0.35%) |
| Mean ± SD | 4.44 ± 0.71 | 3.84 ± 0.88 |

| (d) | **Didactic Value** | |
| --- | --- | --- |
| Rating | DeepSeek-R1 | ChatGPT-o1 |
| 5 | 353 (62.28%) | 88 (15.52%) |
| 4 | 169 (29.80%) | 162 (28.57%) |
| 3 | 42 (7.41%) | 234 (41.26%) |
| 2 | 2 (0.35%) | 74 (13.05%) |
| 1 | 1 (0.18%) | 9 (1.59%) |
| Mean ± SD | 4.54 ± 0.66 | 3.43 ± 0.96 |

**Table S3. (a)-(i)** Grading of the performance of two Large Language Models (DeepSeek-R1 and ChatGPT-o1) in text-based questions across nine evaluation criteria. With seven radiology residents grading 27 text-based questions (knowledge-based and diagnostic / image-description based) there are 189 ratings per criteria. Values represent the absolute cumulative number of ratings (Likert scores 1–5), shown as count (percentage).

| (a) | **Correctness** | |
| --- | --- | --- |
| Rating | DeepSeek-R1 | ChatGPT-o1 |
| 5 | 132 (69.84%) | 98 (51.85%) |
| 4 | 33 (17.46%) | 47 (24.87%) |
| 3 | 17 (8.99%) | 30 (15.87%) |
| 2 | 3 (1.59%) | 10 (5.29%) |
| 1 | 4 (2.12%) | 4 (2.12%) |
| Mean ± SD | 4.51 ± 0.88 | 4.19 ± 1.02 |

| (b) | **Completeness** | |
| --- | --- | --- |
| Rating | DeepSeek-R1 | ChatGPT-o1 |
| 5 | 130 (68.78%) | 61 (32.28%) |
| 4 | 37 (19.58%) | 65 (34.39%) |
| 3 | 16 (8.47%) | 42 (22.22%) |
| 2 | 3 (1.59%) | 15 (7.94%) |
| 1 | 3 (1.59%) | 6 (3.17%) |
| Mean ± SD | 4.52 ± 0.84 | 3.85 ± 1.06 |

| (c) | **Precision** | |
| --- | --- | --- |
| Rating | DeepSeek-R1 | ChatGPT-o1 |
| 5 | 128 (67.72%) | 45 (23.81%) |
| 4 | 49 (25.93%) | 64 (33.86%) |
| 3 | 11 (5.82%) | 67 (35.45%) |
| 2 | 0 (0.00%) | 12 (6.35%) |
| 1 | 1 (0.53%) | 1 (0.53%) |
| Mean ± SD | 4.60 ± 0.65 | 3.74 ± 0.91 |

| (d) | **Comprehensibility** | |
| --- | --- | --- |
| Rating | DeepSeek-R1 | ChatGPT-o1 |
| 5 | 127 (67.20%) | 53 (28.04%) |
| 4 | 54 (28.57%) | 86 (45.50%) |
| 3 | 8 (4.23%) | 47 (24.87%) |
| 2 | 0 (0.00%) | 3 (1.59%) |
| 1 | 0 (0.00%) | 0 (0.00%) |
| Mean ± SD | 4.63 ± 0.57 | 4.00 ± 0.77 |

| (e) | **Clinical Usefulness** | |
| --- | --- | --- |
| Rating | DeepSeek-R1 | ChatGPT-o1 |
| 5 | 106 (56.08%) | 45 (23.81%) |
| 4 | 58 (30.69%) | 62 (32.80%) |
| 3 | 23 (12.17%) | 64 (33.86%) |
| 2 | 1 (0.53%) | 17 (8.99%) |
| 1 | 1 (0.53%) | 1 (0.53%) |
| Mean ± SD | 4.41 ± 0.76 | 3.70 ± 0.94 |

| (f) | **Trustworthiness** | |
| --- | --- | --- |
| Rating | DeepSeek-R1 | ChatGPT-o1 |
| 5 | 76 (40.21%) | 46 (24.34%) |
| 4 | 92 (48.68%) | 74 (39.15%) |
| 3 | 17 (8.99%) | 58 (30.69%) |
| 2 | 3 (1.59%) | 10 (5.29%) |
| 1 | 1 (0.53%) | 1 (0.53%) |
| Mean ± SD | 4.26 ± 0.73 | 3.81 ± 0.87 |

| (g) | **Explanation Depth** | |
| --- | --- | --- |
| Rating | DeepSeek-R1 | ChatGPT-o1 |
| 5 | 125 (66.14%) | 6 (3.17%) |
| 4 | 51 (26.98%) | 56 (29.63%) |
| 3 | 13 (6.88%) | 92 (48.68%) |
| 2 | 0 (0.00%) | 30 (15.87%) |
| 1 | 0 (0.00%) | 5 (2.65%) |
| Mean ± SD | 4.59 ± 0.62 | 3.15 ± 0.81 |

| (h) | **Structure** | |
| --- | --- | --- |
| Rating | DeepSeek-R1 | ChatGPT-o1 |
| 5 | 125 (66.14%) | 57 (30.16%) |
| 4 | 51 (26.98%) | 50 (26.46%) |
| 3 | 13 (6.88%) | 69 (36.51%) |
| 2 | 0 (0.00%) | 11 (5.82%) |
| 1 | 0 (0.00%) | 2 (1.06%) |
| Mean ± SD | 4.59 ± 0.62 | 3.79 ± 0.98 |

| (i) | **Learning Facilitation** | |
| --- | --- | --- |
| Rating | DeepSeek-R1 | ChatGPT-o1 |
| 5 | 103 (54.50%) | 25 (13.23%) |
| 4 | 67 (35.45%) | 56 (29.63%) |
| 3 | 16 (8.47%) | 73 (38.62%) |
| 2 | 2 (1.06%) | 33 (17.46%) |
| 1 | 1 (0.53%) | 2 (1.06%) |
| Mean ± SD | 4.42 ± 0.74 | 3.37 ± 0.95 |

**Table S4.** Comparison of Junior and Senior Resident Ratings for DeepSeek-R1 Across All Evaluation Criteria, including Holm-corrected Mann-Whitney U Test Results.

| DeepSeek-R1 | Junior Residents (n = 4) | Senior Residents (n = 3) | Mann-Whitney-U Test with Holm Correction |
| --- | --- | --- | --- |
| Rating Criteria | Mean ± SD | Mean ± SD | P-Value |
| Overall (all criteria) | 4.50 ± 0.46 | 4.52 ± 0.43 | 1.00 |
| Factual Accuracy | 4.51 ± 0.41 | 4.44 ± 0.43 | .49 |
| - Correctness | 4.62 ± 0.32 | 4.58 ± 0.42 | 1.00 |
| - Completeness | 4.43 ± 0.44 | 4.42 ± 0.32 | 1.00 |
| - Precision | 4.47 ± 0.44 | 4.33 ± 0.50 | 1.00 |
| Clinical Practicality | 4.52 ± 0.43 | 4.64 ± 0.41 | .33 |
| - Comprehensibility | 4.56 ± 0.36 | 4.64 ± 0.26 | 1.00 |
| - Clinical Usefulness | 4.45 ± 0.62 | 4.62 ± 0.62 | 1.00 |
| - Trustworthiness | 4.55 ± 0.25 | 4.65 ± 0.25 | 1.00 |
| Didactic Value | 4.46 ± 0.54 | 4.49 ± 0.44 | 1.00 |
| - Explanation Depth | 4.44 ± 0.72 | 4.61 ± 0.55 | 1.00 |
| - Structure | 4.27 ± 0.49 | 4.26 ± 0.41 | 1.00 |
| - Learning Facilitation | 4.66 ± 0.24 | 4.59 ± 0.25 | 1.00 |

*: significant (p < 0.050); **: p < 0.010; n.s.: p > 0.050

**Table S5.** Comparison of Junior and Senior Resident Ratings for ChatGPT-o1 Across All Evaluation Criteria, including Holm-corrected Mann-Whitney U Test Results.

| ChatGPT-o1 | Junior Residents (n = 4) | Senior Residents (n = 3) | Mann-Whitney-U Test with Holm Correction |
| --- | --- | --- | --- |
| Rating Criteria | Mean ± SD | Mean ± SD | P-Value |
| Overall (all criteria) | 3.81 ± 0.64 | 3.63 ± 0.65 | 0.02 |
| Factual Accuracy | 3.67 ± 0.57 | 3.51 ± 0.58 | 0.49 |
| - Correctness | 3.80 ± 0.54 | 3.67 ± 0.59 | 1.00 |
| - Completeness | 3.44 ± 0.52 | 3.27 ± 0.55 | 1.00 |
| - Precision | 3.79 ± 0.60 | 3.59 ± 0.55 | 1.00 |
| Clinical Practicality | 3.70 ± 0.68 | 3.46 ± 0.69 | 0.12 |
| - Comprehensibility | 3.24 ± 0.48 | 3.02 ± 0.52 | 1.00 |
| - Clinical Usefulness | 3.90 ± 0.77 | 3.78 ± 0.75 | 1.00 |
| - Trustworthiness | 3.95 ± 0.51 | 3.57 ± 0.56 | 0.12 |
| Didactic Value | 4.06 ± 0.58 | 3.93 ± 0.59 | 0.49 |
| - Explanation Depth | 4.18 ± 0.78 | 4.21 ± 0.66 | 1.00 |
| - Structure | 4.00 ± 0.49 | 3.57 ± 0.51 | 0.04 |
| - Learning Facilitation | 4.00 ± 0.43 | 4.00 ± 0.39 | 1.00 |

**Table S6. Accumulated** Distribution of ChatGPT-o1 Ratings Across All Criteria for Text- and Image-based Questions.

| ChatGPT-o1 | Overall rating accumulated across all criteria | |
| --- | --- | --- |
| Rating | Text-based questions | Image-based questions |
| 5 | 436 (25.64%) | 81 (21.43%) |
| 4 | 560 (32.92%) | 112 (29.63%) |
| 3 | 542 (31.87%) | 53 (14.02%) |
| 2 | 141 (8.29%) | 62 (16.40%) |
| 1 | 22 (1.29%) | 70 (18.52%) |
| Mean ± SD | 3.73 ± 0.98 | 3.19 ± 1.42 |

**Table S7.** Overall Intraclass Correlation Coefficients (ICC) for each rating criterion and dimension for all 33 questions (n = 27 text-based, n = 6 image-based) questions across both LLMs with ICC(2) for single-rater and ICC(2,k) for mean rating reliability across seven raters.

| **Rating Criteria** | ICC (2) | ICC (2,k) |
| --- | --- | --- |
| All questions | 0.530 | 0.888 |
| **Factual Accuracy** | 0.608 | 0.916 |
| Correctness | 0.600 | 0.913 |
| Completeness | 0.603 | 0.914 |
| Precision | 0.363 | 0.799 |
| **Clinical Practicality** | 0.435 | 0.844 |
| Comprehensibility | 0.216 | 0.659 |
| Clinical Usefulness | 0.374 | 0.807 |
| Trustworthiness | 0.497 | 0.874 |
| **Didactic Value** | 0.445 | 0.849 |
| Explanation Depth | 0.537 | 0.890 |
| Structure | 0.245 | 0.694 |
| Learning Facilitation | 0.392 | 0.818 |

**
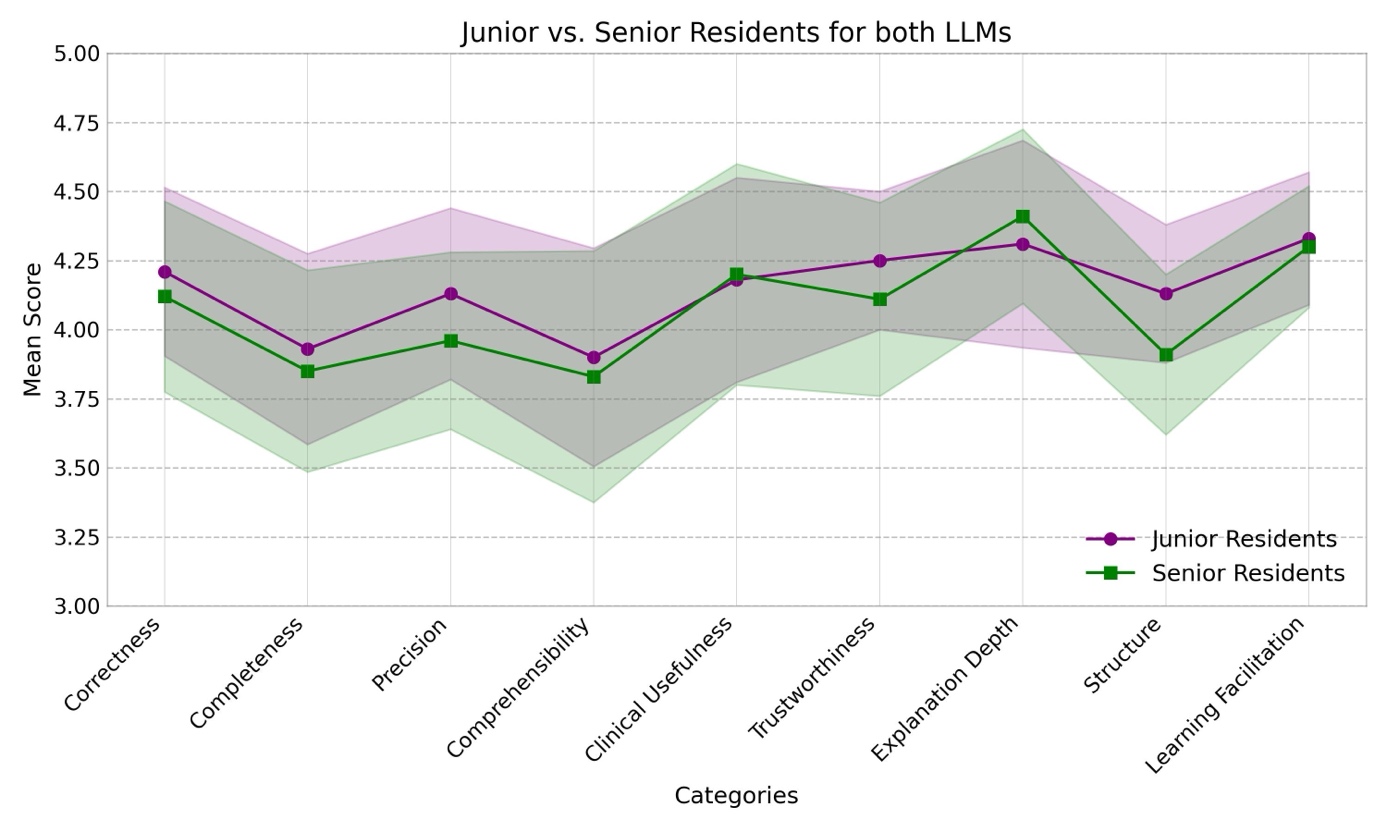
**

**Figure S1.** Line plot comparing the mean ratings of Junior and Senior Residents across every evaluation criterion.
